# Supplementary material for: Characterization of a profilin-like protein from Fasciola hepatica
Source: PeerJ. 2020 Dec 7;8:e10503. doi: 10.7717/peerj.10503 (PMC7727368; doi:10.7717/peerj.10503)
Supplement: Supplemental Information 1 — The amino acid sequences for the different profilins were aligned using the MAFFT program (Katok et al., 2019). The red letters indicate the profilin family domain. White triangles and # indicate phosphatidylinositol phosphate interaction sites and proline binding site respectively. The gray shaded box represents the Apicomplexa parasite specific acid loop motif. The cyan shaded box represents residues involved in actin binding. [file peerj-08-10503-s001.pdf]

```
1          11         21         31         41         50
|          |          |          |          |          |
MS-E-EWCAR CRS-HVGDYK SLASLAFTGL DGNLYGNSDS TN--------   39 F. hepatica_D915_008168
MS-D-AWSQQ CQQ-YLAKYP ALKCLAITSL DGTIYGNSDA TE--------   39 C. sinensis_GAA56606.1
MS-D-AWTQQ CQQ-YLTKYP ALKCLAITSL DGTIYGNSDA TE--------   39 O. viverrini_XP_009173283.1
MSAD-SWDNH CVT-YVANNK CLKNLCMTAI DGSHLGTSNP D---------   39 S. japonicum_CAX7805.1
MA---GWNAY IDN-LMADGT CQDAAIVGYK DSPSVWAAVP GK--------   38 H. sapiens_P07737
MA---GWNAY IDN-LMADGT CQDAAIVGYK DSPSVWAAVP GK--------   38 B. taurus_P02584
M----SWQAY TDN-LIGTGK VDKAVIYSRA GDAVWATSGG LS--------   37 S. cerevisiae_P07274
MAEEYSWDSY LNDRLLATNQ VSGAGLASEE DGVVYACVAQ GEESDPNFDK   50 P. falciparum_P86294
MS---DWDPV VKEWLVDTGY CCAGGIANAE DGVVFAAAAD DD------DG   41 T. gondii_Q58NA1
MS---DWDPV VKEWLVDTGY CCAGGIANAE DGVVFAAAAD DD------DG   41 N. caninum_F0V772
MA---DWVPT IKQLALADNA CYGCGIANAE DGELFSAADI DH------DD   41 B. bovis_Q307P5
      #                              #

---------- ---------- ------FPVT VEFAKSAIAA FSGASPSSLT   63 F. hepatica_D915_008168
---------- ---------- ------FPLD QAFMKQVFAS MKGDPASSIP   63 C. sinensis_GAA56606.1
---------- ---------- ------FPLD QAFMKQVFAS MKGDPASSIP   63 O. viverrini_XP_009173283.1
---------- ---------- ------FRIP PELILQLKSI LDGGLDTSIF   63 S. japonicum_CAX7805.1
---------- ---------- -TFVNITPAE VGVLVGKDR- -SSFYVNGLT   65 H. sapiens_P07737
---------- ---------- -TFVNITPAE VGILVGKDR- -SSFFVNGLT   65 B. taurus_P02584
---------- ---------- -----LQPNE IGEIVQGFDN PAGLQSNGLH   62 S. cerevisiae_P07274
--WSLFYKED YDIEV--EDN GTKTTKTINE GQTILVVFN- -EGYAPDGVW   94 P. falciparum_P86294
--WSKLYKDD HEEDTIGEDG NACGKVSINE ASTIKAAVD- -DGSAPNGVW   87 T. gondii_Q58NA1
--WSKLYKED HEEDTIGEDG NVNGKVTVNE ASTIKAAVD- -DGSAPNGVW   87 N. caninum_F0V772
LCWDSVYRDP YEFEAT-DEN GQPIKHQITE KATIMEVFE- -KRRSSIGIF   88 B. bovis_Q307P5

LNGEKFIVLQ KNDECLIGKC GRKT------ ---------LF VYPCKSSCIF   99 F. hepatica_D915_008168
LAGEKYMCLR SSPECWLGRK EKKA------ ---------IF VYPCKTIAVV   99 C. sinensis_GAA56606.1
LAGEKYMCLR SSPDCWLGRK EKKA------ ---------IF VYPCRTIAVV   99 O. viverrini_XP_009173283.1
FMGEKYIVLQ HDSSCLVSRC GKKS------ ---------LI FYATRKICLV   99 S. japonicum_CAX7805.1
LGGQKCSVIR DSL---LQDG EFSMDLRTKS TGGAPTFNVT VTKTDKTLVL  112 H. sapiens_P07737
LGGQKCSVIR DSL---LQDG EFTMDLRTKS TGGAPTFNIT VTMTAKTLVL  112 B. taurus_P02584
IQGQKFMLLR ADDRSIYGRH DAEG------ ---------VV CVRTKQTVII   98 S. cerevisiae_P07274
LGGTKYQFIN IERDLEFEGY NFDVATCAKL KGGLH---LV KVPGGNILVV  141 P. falciparum_P86294
IGGQKYKVVR PEKGFEYNDC TFDITMCARS KGGAH---LI KTPNGSIVIA  134 T. gondii_Q58NA1
IGGQKYKVVR PEKGFEYNDC TFDITMCARS KGGAH---LI KTPNGSIVIA  134 N. caninum_F0V772
IGGNKYTFAN YDDDCPVGDY TFKCVSAAKN KGGAH---LV KTPGGYIVIC  135 B. bovis_Q307P5
         Δ                  Δ Δ

GLSVDTDESM NATNGNAACA MLSEEYKKLG Y                     130 F. hepatica_D915_008168
GMSQDTESAN NTSNGSDSVA RLAELYMKSN Y                     130 C. sinensis_GAA56606.1
GLSQDTESAN NTSNGSDSVA RLAELYMKSN Y                     130 O. viverrini_XP_009173283.1
GQTVDDDQ-N NCTQGNFAIS RMRDHYERMG Y                     129 S. japonicum_CAX7805.1
LMGKEGVH-- -GGLINKKCY EMASHLRRSQ Y                     140 H. sapiens_P07737
LMGKEGVH-- -GGMINKKCY EMASHLRRSQ Y                     140 B. taurus_P02584
AHYPPTVQ-- -AGEATKIVE QLADYLIGVQ Y                     126 S. cerevisiae_P07274
LYDEEKEQ-- DRGNSKIAAL TFAKELAESS Q                     170 P. falciparum_P86294
LYDEEKEQ-- DKGNSRTSAL AFAEYLHQSG Y                     163 T. gondii_Q58NA1
LYDEEKEQ-- DKGNSRTSAL AFAEYLHQSG Y                     163 N. caninum_F0V772
VFDENRGQ-- NKTASRMAAF ALAEYMAANG Y                     164 B. bovis_Q307P5
                  Δ       #  Δ   #
```
