# Supplementary material for: Characterization of a profilin-like protein from Fasciola hepatica
Source: PeerJ. 2020 Dec 7;8:e10503. doi: 10.7717/peerj.10503 (PMC7727368; doi:10.7717/peerj.10503)
Supplement: Supplemental Information 2 [file peerj-08-10503-s002.pdf]

Figure 2

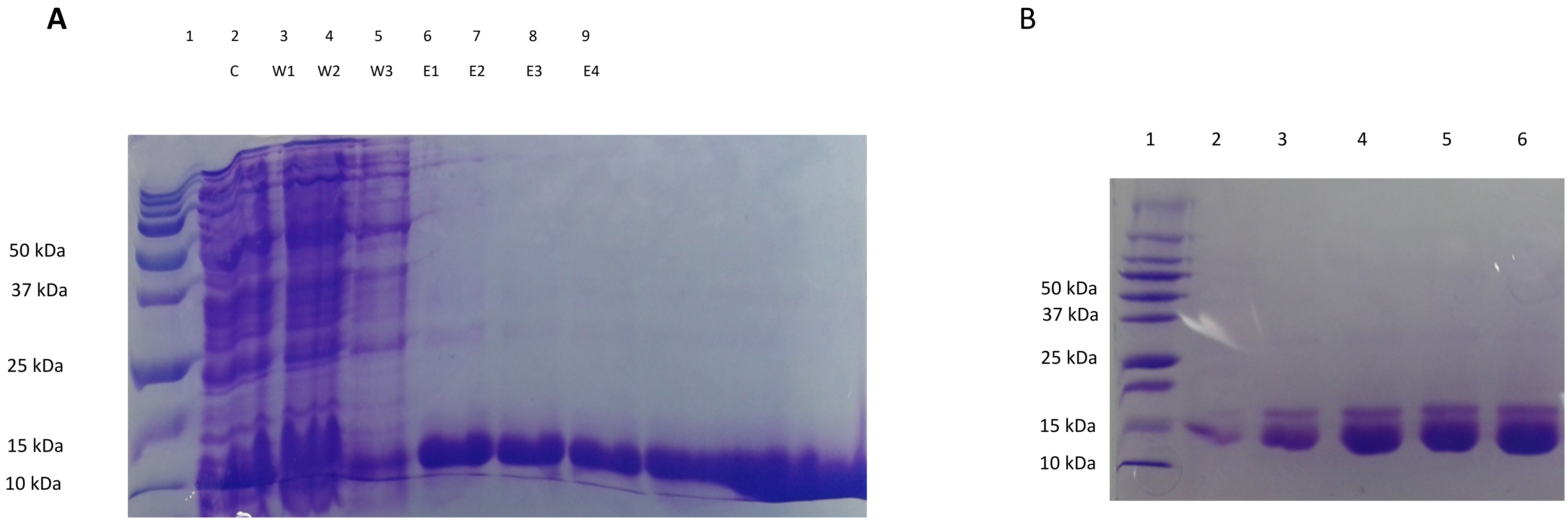

Figure 2. Recombinant expression and purification of FhProfilin. A) Cell lysate expressing FhProfilin were applied to a NI-IDA column and washed twice before elution with imidazole. 15 µl of each stage of the purification was resolved by SDS-PAGE and stained with Coomassie blue. C = starting material, W1 = wash 1, W2=wash 2, E1,2,34 = elution fractions B) Size-exclusion chromatography fractions (68,70,72,74,76) was resolved by SDS-PAGE and stained with Coomassie blue.

Figure 3

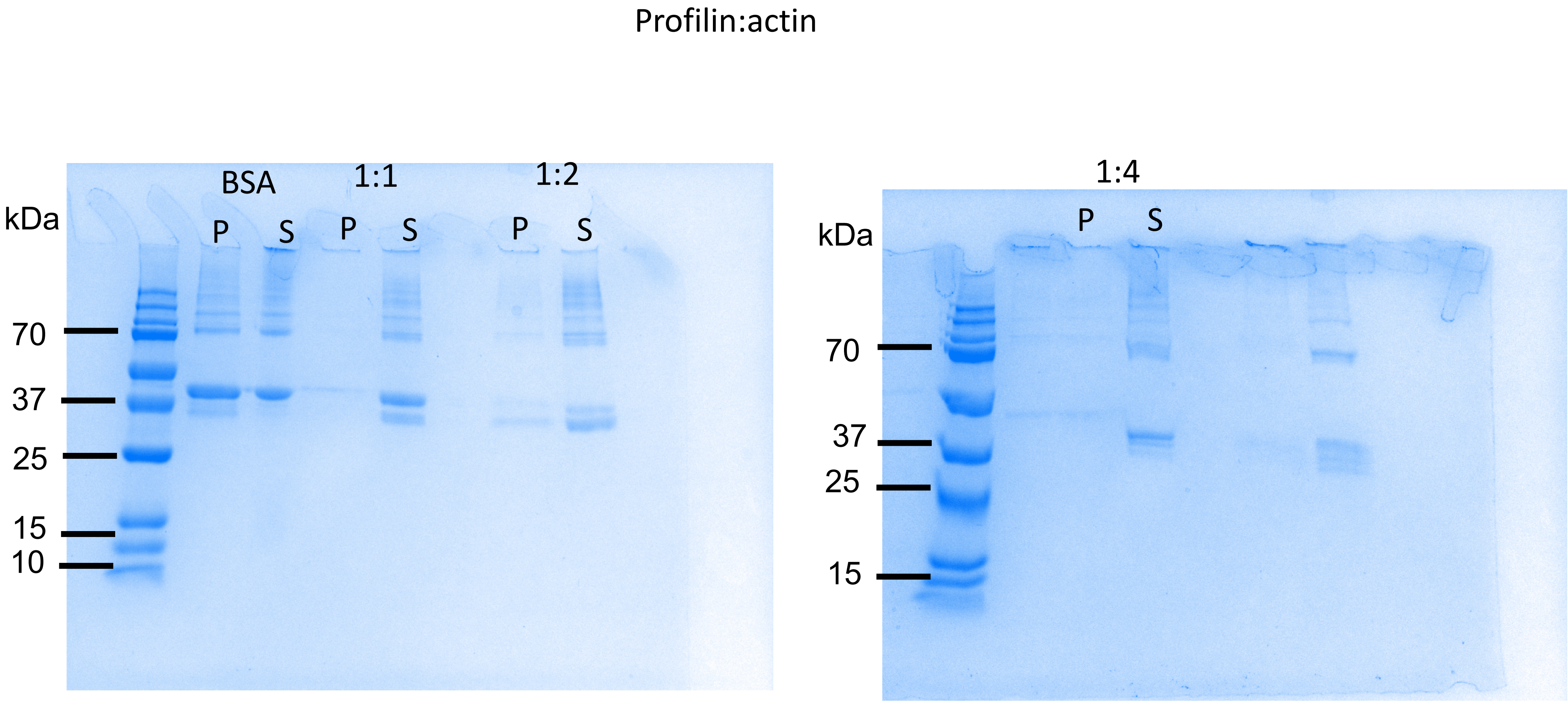

SDS-PAGE analysis of polymerised actin incubated with FhProfilin. Different ratios of FhProfilin and actin were incubated and separated into polymerised or monomeric actin fractions by centrifugation. The pellet (p) and supernatant (s) fractions (15 µL) was resolved by SDS-PAGE and stained with Coomassie blue.

Figure 5

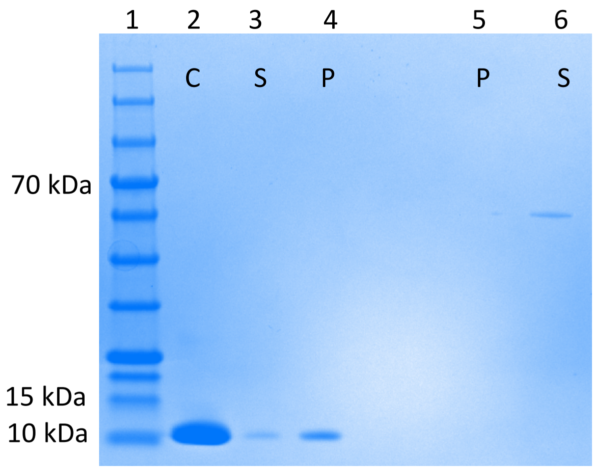

SDS-PAGE analysis the ploy-L-proline affinity assay. The second lane is unbound, SEC purified FhProfilin, used as a positive control. Lanes 3 and 4 are the supernatant and pellet of the test samples respectively. Lanes 5 and 6 are the pellet and protein samples of proline incubated with BSA respectively.

Figure 6

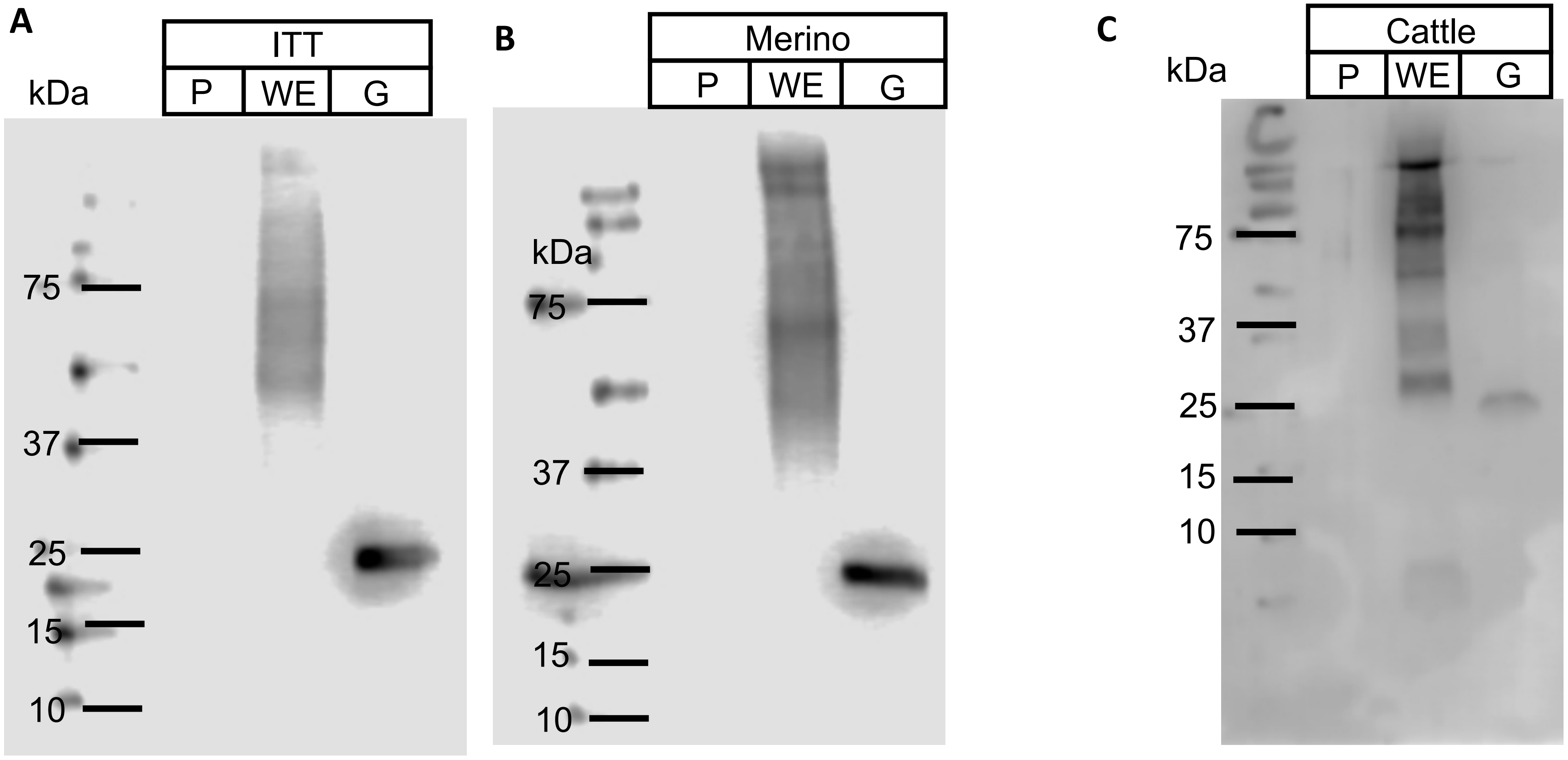

Figure 6. **Western blot analysis of FhProfilin with immune sera.** Immune sera from infected ITT (A), Merino (B) and cattle (C) was probed on whole fluke extract (WE), recombinant *Fh*Profilin (P), purified native *Fh*GST (G).
